# Targeting regulatory T cells for cardiovascular diseases

**DOI:** 10.3389/fimmu.2023.1126761

**Published:** 2023-02-23

**Authors:** Xinting Wang, Hua Zhou, Qian Liu, Peipei Cheng, Tingyao Zhao, Tianshu Yang, Yue Zhao, Wanjing Sha, Yanyan Zhao, Huiyan Qu

**Affiliations:** ^1^ Institute of Cardiovascular Disease of Integrated Traditional Chinese and Western Medicine, Shuguang Hospital Affiliated to Shanghai University of Traditional Chinese Medicine, Shanghai, China; ^2^ Department of Cardiovascular Disease, Shuguang Hospital Affiliated to Shanghai University of Traditional Chinese Medicine, Shanghai, China; ^3^ Guanghua Hospital Affiliated to Shanghai University of Traditional Chinese Medicine, Shanghai, China; ^4^ Shuguang Hospital Affiliated to Shanghai University of Traditional Chinese Medicine, Shanghai, China

**Keywords:** regulatory T cells, cardiovascular diseases, immune balance, inflammation, cardiac remodeling, immune tolerance, cardiac regeneration

## Abstract

Cardiovascular diseases (CVDs) are the leading cause of death and disability worldwide. The CVDs are accompanied by inflammatory progression, resulting in innate and adaptive immune responses. Regulatory T cells (Tregs) have an immunosuppressive function and are one of the subsets of CD4^+^T cells that play a crucial role in inflammatory diseases. Whether using Tregs as a biomarker for CVDs or targeting Tregs to exert cardioprotective functions by regulating immune balance, suppressing inflammation, suppressing cardiac and vascular remodeling, mediating immune tolerance, and promoting cardiac regeneration in the treatment of CVDs has become an emerging research focus. However, Tregs have plasticity, and this plastic Tregs lose immunosuppressive function and produce toxic effects on target organs in some diseases. This review aims to provide an overview of Tregs’ role and related mechanisms in CVDs, and reports on the research of plasticity Tregs in CVDs, to lay a foundation for further studies targeting Tregs in the prevention and treatment of CVDs.

## 1 Introduction

Regulatory T cells (Tregs), CD4^+^CD25^+^Foxp3^+^Tregs, secrete anti-inflammatory factors such as interleukin (IL)-10 and transforming growth factor-β (TGF-β), which have immunosuppressive effects (1). Tregs account for 5 ~ 10% of all CD4^+^ T cells. There are two sources of Tregs: derived from the normal thymus (natural Tregs, nTregs); Or derived from peripheral naive CD4^+^ T cells induced to differentiate into Tregs (inducible Tregs, iTregs). Foxp3 is a specific marker of Tregs, and an essential regulator of Tregs development and function (2). In comparison, the absence of Foxp3 will lead to the loss of Treg function, which is closely associated with severe autoimmune diseases in humans (3) and rodents (4). Tregs play a key role in immune dynamic balance (5) and regulate immunity in Corona Virus Disease 2019 (COVID-19) (6), tumors (7), infectious diseases (8), and transplant rejection (9, 10).

Cardiovascular diseases (CVDs) are the leading cause of death and disability worldwide, with the number of people affected by CVDs increasing from 271 million in 1990 to 523 million in 2019 (11). Inflammation plays a vital role in many CVDs, disrupting the immune balance of the body and causing innate and adaptive immune responses. Tregs can prevent the progression of CVDs by regulating immunity (12). In this review, we summarized the role and related mechanisms of Tregs in the prevention and treatment of CVDs, mainly reflected in the regulation of immune balance, inflammation, cardiac and vascular remodeling, immune tolerance, and cardiac regeneration. CVDs cover heart failure (HF), myocardial infarction (MI), myocarditis, atherosclerosis, hypertension, and atrial fibrillation. The research progress and clinical potential of targeted Tregs therapy for CVDs are further elaborated.

## 2 Common CVDs and their epidemiology

### 2.1 Heart failure

2022 AHA/ACC/HFSA defines HF as a complex clinical syndrome with symptoms and signs that result from any structural or functional impairment of ventricular filling or ejection of blood (13). The latest data for 2021 show that the number of HF patients worldwide has increased from 33.5 million in 1990 to 64.3 million in 2017 (14). Up to 25% ~ 40% of patients die of Chronic HF one year after being diagnosed with HF (15, 16). HF is a leading cause of death, affecting more than 24 million people worldwide (17). HF is a significant public health problem in the world with high incidence, re-hospitalization, disability, and mortality (18). The occurrence and development of HF are accompanied by activation and inflammation of the immune system (19), and the immune system regulates inflammation by secreting related factors.

### 2.2 Myocardial infarction

MI is ischemic necrosis of the myocardium caused by transient or persistent occlusion of the distal coronary artery, associated with high morbidity and mortality, resulting in more than 15 million deaths worldwide every year (20, 21). Due to different medical conditions, MI prevalence varies widely among regions, ranging from 3 to 20% (22–24). Although coronary revascularization treatment strategies can reduce MI mortality (25), MI is associated with inflammation, cardiac remodeling, myocardial fibrosis, and other pathological processes (21, 26), which aggravate clinical cardiovascular malignant events. The immune system plays a critical role in the occurrence and development of post-MI inflammation (27).

### 2.3 Myocarditis

Myocarditis is an inflammatory disease of myocardium cells with a broad range of clinical and histological manifestations of cardiac pathological immune processes that can lead to acute HF, sudden death, and chronic dilated cardiomyopathy (28, 29). Myocarditis can be attributed to immune responses, viral infections, and bacterial infections. Myocarditis includes periods of acute inflammation, subacute inflammation, and myopathy, resulting in cardiac remodeling, myocardial fibrosis, and cardiac dysfunction (30, 31). Cardiac magnetic resonance imaging (MRI) and molecular detection of viruses by endomyocardial biopsy are effective methods for the clinical diagnosis of myocarditis. However, it is difficult to sample human heart tissue, and it is necessary to explore the pathophysiological mechanisms in experimental animal models (32, 33). Experimental autoimmune myocarditis (EAM) induced by myocardial myosin is a classic model of autoimmune myocarditis (34). Viral myocarditis (VMC) caused by Coxsackievirus B3 (CVB3) infection is the main cause of sudden cardiac death in the young population (30). VMC is characterized by immune-mediated inflammation of the myocardium caused by viral infection (35).

Chronic Chagas disease cardiomyopathy (CCC), progressive inflammation of the heart caused by *Trypanosoma cruzi* (*T.cruzi*) infection, manifesting as diffuse myocardial fibrosis, cardiac hypertrophy, myocardial injury, progression to HF, and death, has become a major public health disease in Latin America (36). Chagas’ etiology of HF has become the third most common indication for heart transplantation in South America (37). Parasite-dependent myocardial aggression and immune-mediated tissue damage are key pathological mechanisms of CCC (38, 39). Therefore, targeted modulation of immunity has become a strategy for the treatment of CCC (40).

### 2.4 Hypertension

The number of hypertensive patients worldwide has grown from 128 million in 1990 to 650 million in 2019, and more than 700 million were unaware of their hypertension status (41). Hypertension is an important risk factor for CVDs, which significantly increases the incidence of coronary heart disease and HF complications. Hypertension is an inflammatory disease, and the inflammatory markers C-reactive protein (CRP), various cytokines, and pathway complement pathway products are increased in patients with hypertension (42).

### 2.5 Atherosclerosis

In 2020, nearly 2 billion people worldwide suffer from carotid atherosclerosis, which increases the risk of coronary heart disease events (43). In the general middle-aged population, 42.5% had silent coronary atherosclerosis and 5.2% had severe atherosclerosis (coronary significant stenosis ≥50%) (44). Atherosclerosis is a chronic inflammatory disease of the vascular wall which involves cellular immune responses (45). Acute and chronic myocardial ischemia caused by coronary atherosclerosis is the most common cause of HF, and studies have shown that Tregs have atherosclerotic protective effects (46, 47).

## 3 Tregs-related membrane molecules

### 3.1 CD4/CD25

T cells specifically recognize antigens presented by antigen-presenting cells (APCs) through T cell receptors (TCRs), and recognize antigens through CD3 molecular transduction, forming TCR-CD3 complexes, generating activation signals, and transmitting them to cells. CD4 recognizes and binds MHC-II molecules, and CD4^+^T cells specifically recognize exogenous antigens presented by MHC-II molecules. Tregs highly express IL-2 receptor α (IL-2Rα, CD25), It promotes the binding of IL-2 and CD25 without binding with other receptors. It is called CD25-biased IL-2 antibody complexes, which promote the activation and proliferation of Tregs (48). Tregs highly express the high-affinity receptor for IL-2 and competitively prey on IL-2 that is required for the survival of neighboring activated T cells, resulting in suppressed proliferation, followed by apoptosis, of activated T cells.

### 3.2 CD28

CD28 is a homodimer composed of two identical peptide chains, which is expressed in 90% of CD4^+^T cells. The costimulatory signal produced by CD28 plays an important role in the activation of T cells. Gain of Tregs function was accomplished by therapeutic administration of superagonistic CD28-specific monoclonal antibodies (CD28-SA) that preferentially activate Tregs over conventional CD4^+^ T cells *in vivo* due to a vigorous co-stimulatory signal induced by cross-linking of CD28 molecules (49, 50). CD28 super-agonists, which effectively target Tregs, hold great promise for the treatment of human autoimmune diseases (51).

### 3.3 CTLA4

Tregs constitutively express the inhibitory receptor cytotoxic T-lymphocyte associated protein 4 (CTLA4, CD152). The cytoplasmic region of CTLA4 has immunoreceptor tyrosine-based inhibitory motifs (ITIMs), which transmit inhibitory signals. Human CTLA-4 haploinsufficiency caused dysregulation of Foxp3^+^ Tregs, hyperactivation of effector T cells, and lymphocytic infiltration of target organs (52). Deletion of CTLA-4 in mice impairs Tregs’ suppressive function, causing severe autoimmune disease and early lethality, despite normal Foxp3 levels (53, 54).

### 3.4 PD-1

Programmed cell death protein 1(PD-1, CD279) is a Treg surface costimulatory marker molecule with ligands programmed cell death ligand 1 (PD-L1) and PD-L2. PD-1, when bound to its ligands, can inhibit the proliferation of effector T cells and activated B cells. Furthermore, PD-L1-Ig induced Naïve CD4^+^ T cells to differentiate, proliferate into CD4^+^Foxp3^+^Tregs, and enhanced the immunosuppressive function of Tregs (55, 56).

### 3.5 CD39/CD73

Ectonucleoside triphosphate diphosphohydrolase-1 (ENTPD1, CD39) and ecto-5 ′-nucleotidase (e5NT, CD73) are expressed on the surface of Tregs. CD39 degrades adenosine triphosphate (ATP) into adenosine diphosphate (ADP)/adenosine monophosphate (AMP), CD73 degrades ADP/AMP into adenosine, and the CD39/CD73 pathway converts pro-inflammatory ATP into adenosine with anti-inflammatory properties, which further exerts immunosuppressive functions and inhibits the activation of T cells and the production of inflammatory mediators (57, 58). CD73 deficiency reduces cardiac chemotaxis of Tregs, impairing the immunosuppressive and protective functions of Tregs during cardiac healing (59). Increased Foxp3 nuclear levels and enhanced CD39 and CD73 transcription in NADPH oxidase 2 (NOX2) KO Tregs effectively inhibit effector T cell proliferation and reverse angiotensin (Ang) II-induced cardiac remodeling (60).

## 4 Tregs regulate immune balance in CVDs

### 4.1 Th17/Treg

Naive CD4^+^ T cells differentiate into different subsets of cells according to different cytokine environments, including 1 helper T (Th1)cells, Th2 cells, Th17 cells (61), and Tregs, which share the exact origin but exhibit opposite effects (62). Th17 cells express the transcription factor retinoid-related orphan receptor-γt (RORγt). Th17 cells, characterized by the production of IL-17, contribute to fibrosis and fibrotic diseases (63), induce autoimmunity, and promote inflammation (64). IL-17 activates the protein kinase C (PKC)β/extracellular signal-regulated kinase 1/2 (ERK1/2)/nuclear factor-κB (NF-κB)-dependent signaling pathway to aggravate the degree of myocardial fibrosis (65). IL-17 activates the MAPK pathway and increases the expression of downstream target genes IL-6, tumor necrosis factor (TNF), C-C Motif Chemokine Ligand (CCL) 20, and C-X-C Motif Chemokine Ligand (CXCL) 1 to worsen cardiac remodeling (66). The microRNA mmu-miR-721, synthesized by Th17 cells, was present in the plasma of mice with acute autoimmune or viral myocarditis, but not in those with AMI. And the human homolog (hsa-miR-Chr8:96) is a novel microRNA that distinguishes myocarditis patients from MI patients (67). Tregs inhibit inflammation and regulate immune balance by secreting IL-10 and TGF-β (1, 68). IL-10 is a key anti-inflammatory mediator. IL-10 treatment significantly improves the left ventricular dilation and ejection fraction of MI mice, promotes the polarization of M2 macrophages to reduce cardiac inflammation, activates fibroblasts to reduce extracellular matrix collagen deposits, and promotes cardiac healing and improves cardiac remodeling (69). However, IL-10 gene deletion enhanced neutrophil infiltration, increased inflammation, enlarged myocardial infarction area, and myocardial necrosis in ischemia-reperfusion mice (70). TGF-β is a crucial enforcer of immune homeostasis and tolerance, and plays an important role in cell development, differentiation, inflammation, and tissue repair (71). However, TGF-β1 gene deletion results in nearly 50% mouse embryonic lethality, with mice born with uncontrolled inflammation and dying at 3-4 weeks (72, 73). Th17/Treg maintains immune dynamic equilibrium when the number and function of Th17 cells and Tregs are balanced.

Clinically, increased Th17 cells ratio and decreased Tregs ratio lead to pathological manifestations of Th17/Treg immune imbalance, which are widely found in patients with cardiac inflammatory diseases such as acute coronary syndrome (74), congestive HF (75), and rheumatic heart disease (76). Serum IL-17 levels of Th17 characteristic cytokine were significantly increased in HF patients, and IL-10 levels of Tregs characteristic cytokine were significantly decreased (75, 77). The Th17/Treg ratio is an independent predictor for 1-year mortality in patients with MI-related cardiogenic shock (78). Th17/Treg ratio combined with CRP level in serum predicts atrial fibrillation after off-pump coronary artery bypass transplantation (79). Moreover, intensive statin therapy improves Th17/Treg functional imbalance in patients with non-ST elevation acute coronary syndromes undergoing percutaneous coronary intervention, reduces cytokines IL-17, IL-6, and IL-23 secreted by Th17 cells, and increases cytokines IL-10 and TGF-β1 secreted by Tregs (80). The pathological phenomenon of Th17/Treg imbalance is widely found in obese children with systolic hypertension (81), patients with resistant hypertension (82), carotid atherosclerotic hypertension (83), and pulmonary hypertension (84). The Th17/Treg imbalance is a vital contributor to the high incidence of atherosclerosis in systemic lupus erythematosus patients (85). In addition, The Th1/Treg ratio and Th17/Treg ratio were significantly increased in patients with rheumatoid arthritis combined with atrial fibrillation, and the increased Th1/Treg ratio was a risk factor for rheumatoid arthritis combined with atrial fibrillation (86).

Mechanistically, in the studies of the ischemic HF model induced by coronary artery ligation in mice (87), and the HF model induced by abdominal aortic ligation in rats (77), it was found that Th17/Treg ratio was increased in failing myocardium. Th17/Tregs imbalance regulates cardiac fibrosis and heart failure in rats by regulating lysyl oxidase (LOX) expression, Th17 cells aggravate fibrosis-related indicators (matrix metalloproteinase-2/matrix metalloproteinase-9 (MMP-2/9) and collagen I/III) and LOX expression by activating the IL-17/ERK1/2-activating protein-1 (AP-1) pathway, while Tregs inhibit fibrosis-related indicators and LOX expression by activating the IL-10/Janus kinase (JAK) 1-signal transducer and activator of transcription (STAT)3 pathway (77). allogeneic skeletal myoblasts transplantation (allo-SMT) is a potential strategy to treat MI. However, the host immune response to donor skeletal myoblasts is intensified, as evidenced by further Th17/Treg imbalance, which reduces the therapeutic effect of allo-SMT. It was confirmed that transfected vascular endothelial-derived growth factor (VEGF) 165 allo-SMT decreased the expression of RORγt, increased the expression of Foxp3, and promoted the Th17/Treg balance in MI (88). Furthermore, aerobic exercise (89), and catechin (90) interventions can significantly reduce the cardiac Th17/Treg ratio in HF model animals, and improve the cardiac function and immune environment. Targeted inhibition of microRNA-155 significantly reduced cardiac Th17 cell infiltration, and Th17 cells related factor (RORγT, IL-17A, IL-6) expression levels decreased in EAM mice. Targeted inhibition of microRNA-155 resulted in increased expression of Th17 cells related proinflammatory factors (RORγT, IL-17A, IL-21, IL-22) in splenic CD4+ T cells of EAM mice and Treg associated anti-inflammatory factors (Foxp3, TGF-β, IL-10, IL-35) were downregulated without affecting Treg function. Therefore, Targeted inhibition of microRNA-155 attenuated myocardial inflammation, mainly inhibiting Th17 cell immune responses, and then adjusted the immune balance of Th17/Treg (91). Fenofibrate intervention (92) can reduce the severity of EAM disease and cardiac injury by regulating Th17/Treg immune response.

Long-term exposure of parents to particulate matter (PM) _2.5_ air pollution may induce increased blood pressure in offspring by mediating an imbalance of the Th17/Treg immune microenvironment (93). Interventions with *Lactobacillus fermentum* CECT5716 (94), fecal microbiota transplantation (95), and *Dieckol (* 96) attenuate Th17/Treg imbalance in the mesenteric lymph nodes and aorta of spontaneously hypertensive rats (SHR), attenuate endothelial cell dysfunction, and control blood pressure. Electroacupuncture effectively reduces systolic blood pressure by promoting SHR Th17/Treg immune balance (97). Inhibition of serum/glucocorticoid regulated kinase 1 (SGK1) can reduce the translocation of factor forkhead box O1 (FoxO1) from the cytoplasm to the nucleus, ameliorate the Th17/Treg imbalance, and target organ damage to the heart and kidney in Ang II-induced hypertension mice (98).

Th17 cells mediate pro-inflammatory responses to exacerbate atherosclerosis, whereas Tregs exert atheroprotective effects by suppressing inflammation and stabilizing plaques (99). Targeting the Th17/Treg balance has emerged as a strategy for the treatment of atherosclerosis (100). Th17/Treg function is imbalanced during high-fat diet-induced atherosclerosis in age and apolipoprotein E (ApoE)^-/-^ mice (101), and *Porphyromonas gingivalis* oral infection further exacerbated Th17/Treg imbalance and atherosclerosis plaque deterioration (102). However, pharmacologic interventions by pioglitazone (103), traditional Chinese medicine AnGong Niuhuang Pill (104), and Yangyin Qingre Huoxue Prescription (105) exert anti-atherosclerotic effects by regulating Th17/Treg balance, inhibiting chronic inflammation, reducing plaque collagen fibers, and stabilizing plaques.

### 4.2 CD4^+^ T cell subsets and Tregs

**Figure 1:**
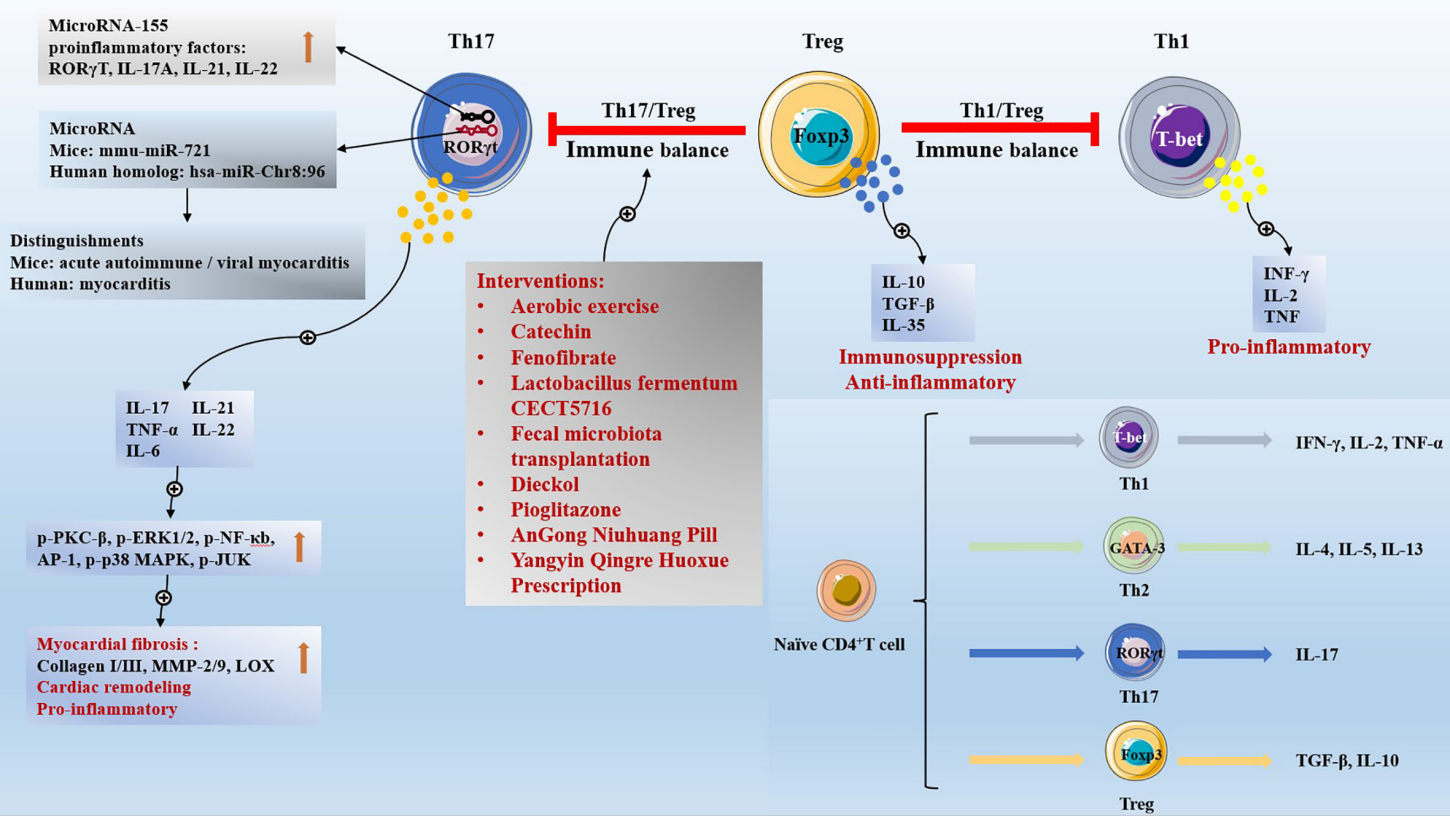
The immune balance involved by Tregs and the differentiation of Naïve CD4+ T cells.

The earliest CD4^+^ T cell subsets to be discovered are Th1 cells and Th2 cells; Secretion of INF- γ, IL-2, and TNF by Th1 cells, and the key transcription factor is T-bet; Th2 cells secrete IL-4, IL-5, and IL-13, and the key transcription factor is GATA-3. Patients with acute coronary syndrome have a decreased proportion of circulating Tregs and an increased proportion of Th1 and Th17 cells. IL-37-treated human dendritic cells acquire a tolerogenic dendritic cells (tDCs) phenotype, with tDCs promoting the expansion of CD4^+^ T cells into Tregs and reducing Th1 and Th17 populations (106). Blocking angiotensin II (AII) production with angiotensin-converting enzyme (ACE), inhibitors or inhibiting AII signal transduction with angiotensin type 1 receptor (AT1R) blockers inhibited self-reactive Th1 and Th17 cells and promoted CD4^+^FoxP3^+^Tregs (107). Cardiac biopsy in patients with dilated cardiomyopathy showed that cardiac T cell infiltration was characterized by differential expression of functional T cell markers, including Th1 markers (IFN-γ, T-bet, Eomesodermin), Tregs (Treg; Foxp3, TGF-β) and cytotoxic T-cells (CTL: Perforin, Granulysin, Granzyme A) increased significantly, while Th17 had no major effect (108). Th1 cells promote inflammation and increase the volume of MI (109). Seven days after MI, the CD4^+^ T cells in the heart of hyaluronan synthase 3 (HAS3) KO mice were significantly reduced, with CD4^+^CXCR3^+^Th1 cells and CD4^+^CD25^+^Tregs (110). Progranulin down-regulates the response of Th1 and Th17 cells and the production of inflammatory cytokines by inhibiting the JAK/STAT pathway, and improving CVB3-induced VMC (111). Nicotine activates the cholinergic anti-inflammatory pathway to reduce the inflammatory response of VMC. Nicotine treatment increases the proportion of Th2 cells and Tregs, reduces the proportion of Th1 and Th17 cells in the spleen, and reduces the myocardial injury and inflammatory cell infiltration of VMC (112). VMC mice vagotomy inhibited the activation of JAK2/STAT3 and enhanced NF-κB in spleen CD4^+^T cells, resulting in an increase in the proportion of Th1 and Th17 cells and a decrease in the proportion of Th2 cells and Tregs in the spleen (113). In atherosclerotic diseases, Th1 plays a pro-inflammatory role while Tregs play an anti-inflammatory role. LCK inhibitor inhibits PP2, inhibits the infiltration of CD4^+^ T cells in plaque, increases Tregs, and reduces the synthesis of TNF-γ And TNF-α by Th1 cells, Inhibition of PI3K/AKT/mTOR signal activation reduces Th1/Treg ratio and plays an anti-atherosclerotic role (114). Ang II treatment of ApoE^-/-^ mice resulted in plaque enlargement and modulation of CD4 T cell subset activity: increased Th1 and Th17 cells; Decreased Th2 cells and Tregs. Valsartan can reduce the systolic pressure of Ang II treated ApoE^-/-^ mice, promote the differentiation of CD4^+^T cells into Th2 cells and Tregs, improve the immune balance, and stabilize the atherosclerosis plaque (115). Allergic asthma accelerated atherosclerosis and was accompanied by increased splenic Th2 and Th17 cells and decreased Tregs. Curcumin treatment for 8 weeks attenuates the aggravation of atherosclerotic lesions and stabilizes plaques by decreasing Th2 and Th17 cells and increasing Tregs, which regulate the balance of Th2/Tregs in asthmatic ApoE^-/-^ mice (116). The immune balance involved by Tregs and the differentiation of Naïve CD4^+^ T cells are shown in **Figure 1**.

## 5 Tregs regulate inflammation in CVDs

### 5.1 Myocardial infarction

MI is a sterile inflammatory response disease with exacerbated inflammation in the heart. MI leads to the death of cardiomyocytes exposed to endogenous damage-associated molecular patterns (DAMPs) of the innate immune system, and damps are recognized by pattern recognition receptors (PRRs), which promote the release of chemokines and proinflammatory cytokines that recruit and activate neutrophils, monocytes, and macrophages to the infarct zone, exacerbating cardiac inflammation. Importantly, the monocytes recruited from the circulation are differentiated into macrophages in the infarct zone, which are monocytes/macrophages (Mos/Mps). Mos/Mps are critical immune cells that determine the progression and repair of inflammation after MI. Macrophages have two phenotypes: M1 Macrophages with pro-inflammatory properties, and M2 Macrophages with anti-inflammatory and repair properties (117–119). Treg promoted the polarization of Mos/Mps to M2 type and improved immune homeostasis and cardiac repair after MI. However, in Treg-depleted mice (Foxp3DTR) MI model mice with Treg ablation and MI model mice with Treg depletion, Mos/Mps polarized to the M1 type and intensified the inflammatory response (120). DCs-derived exosomes activated Tregs-mediated M2 type polarization of macrophages, and significantly increased border zone infiltration of Tregs and M2 macrophages in MI model mice, thereby improving cardiac function (121). As a pro-inflammatory mediator of C-C motif chemokine receptor (CCR) 2^+^ macrophages and DCs, CCL17 inhibits Tregs recruitment by biased activation of CCR4. However, deletion of the CCL17 gene enhanced Tregs recruitment, weakened gene expression of inflammatory macrophages, and improved heart function and cardiac remodeling in MI mice (122). After MI, CXCL12/C-X-C motif chemokine receptor (CXCR) 4 chemotaxis inflammatory cells to the infarct area. CXCR4 antagonist specifically enhanced the recruitment of splenic Treg to the infarct zone by initiating DCs, inhibited the gene expression of pro-inflammatory Mos/Mps, improved cardiac function, and promoted cardiac repair in AMI reperfusion mice (123). Nuclear paraspeckle assembly transcript 1 (NEAT1) is a novel long noncoding RNA (IncRNA) immunomodulator that affects the process of Mos/Mps and T cell differentiation. LncRNA NEAT1 expression is decreased in peripheral blood monocytes of MI patients. Maldifferentiation of Mos/Mps in the bone marrow and blood of NEAT1^-/-^ mice, abnormal differentiation of Tregs in the spleen, increased infiltration of CD68^+^ macrophages in the aortic wall, and imbalance of the immune system (124). In addition, CD73 derived from CD4^+^Foxp3^+^Tregs can bind to CD4^+^Foxp3^-^Teffs and reduce IL-1β, TNF-α, IFN-γand IL-17 levels, suppressing inflammatory responses and protecting against MI (59).

### 5.2 Hypertension

Treg deficiency exacerbates hypertension progression by enhancing innate and adaptive immune responses (125, 126). Depletion of Tregs significantly increased systolic blood pressure (127). Adoptive transfer of Tregs improved hypertension, vasodilatory injury, and immune cell infiltration (128), and inhibited autophagy, oxidative stress, and inflammation to improve hypertensive microvascular function (129). Complement C3a receptor (C3aR) and complement C5a receptor (C5aR) double knockout mediates Tregs function and attenuates Ang II-induced inflammatory cytokine expression, target organ injury, and elevated blood pressure (130). Cystathionine γ lyase-derived hydrogen activates liver kinase B1 (LKB1) and promotes differentiation and proliferation of Tregs, reducing immune inflammation in blood vessels and kidneys, thereby preventing hypertension (128). Doxycycline improves intestinal barrier integrity by reducing *Lactobacillus* and high plasma L-lactate levels, reducing aortic oxidative stress, increasing Tregs infiltration and IL-10, and improving vascular dysfunction and blood pressure in deoxycorticosterone acetate (DOCA)-salt-induced hypertension model rats (131). Activation of the PD-1/PD-L1 pathway significantly increased Tregs ratio and Foxp3 mRNA expression, and increased the levels of anti-inflammatory factors TGF-β, IL-10, and IL-35 in peripheral blood monocytes (PBMC), improving gestational hypertension (132).

### 5.3 Atherosclerosis

Atherosclerosis is a vascular inflammatory disease (133). In CVDs, atherosclerotic lesions can cause cardiac ischemia and lead to infarction. Significantly, the adoptive transfer of Tregs dampens inflammatory responses and protects against atherosclerosis (134, 135). Tregs inhibit effector T cells, induce M2-type polarization of macrophages, and accumulate them in plaques, enhancing inflammation dissipating and plaque regression. During lipid-lowering therapy, Tregs in regressing plaques are peripherally induced and characterized by the lack of Neuropilin 1 (Nrp1) and Helios expression (136). Activation of the Tregs/Indoleamine 2,3-dioxygenase axis forms a tolerant immune environment characterized by reducing vascular inflammation and atherosclerotic lesions (137), which has a protective effect on atherosclerotic CVDs. Overexpression of autophagy related 14 (ATG14) can reverse the autophagy dysfunction of macrophages in ApoE^-/-^ mice plaques, inhibit the accumulation of sequestosome 1 (SQSTM1)/P62, promote the differentiation of Tregs and up-regulate the number of Tregs, and reduce the inflammation and lesions of atherosclerosis (138). Recombinant human IL-37 (139) and traditional Chinese medicine Si-Miao-Yong-An decoction (140) can regulate the immune environment and improve atherosclerotic lesions by reducing inflammatory macrophage infiltration and increasing Tregs. Activation of Tregs/Indoleamine 2,3-dioxygenase axis forms a tolerant immune environment characterized by reducing vascular inflammation and atherosclerotic lesions (137), which has a protective effect on atherosclerotic CVDs. However, Inducible T cell costimulatory (ICOS)^-/-^ (141), CD80^-/-^CD86^-/-^ (135), and hyperhomocysteinaemia (134) can reduce the number of Tregs, suppress the immunosuppressive function, and aggravate the development of atherosclerosis. Tregs depletion exacerbates atherosclerotic lesions, which are associated with hypercholesterolemia caused by abnormal lipoprotein metabolism (142), and exacerbates inflammatory responses by preventing plaque contraction (136).

### 5.4 Experimental autoimmune myocarditis

Single-cell RNA sequencing analysis of CD45^+^ cells extracted from the hearts of EAM model mice revealed that Tregs were the predominant T-cell population detected during the subacute inflammatory phase (143). Extracellular vesicles secreted by human-derived heart stromal/Progenitor cells (144), adenovirus vector-mediated gene transfer of CTLA4 Ig fusion protein (145), CD28 superagonists (146), and Oleanolic Acid (147) interventions can protect the heart function and alleviate inflammation of EAM model rodents by increasing the number of Tregs and enhancing the immunosuppressive function of Tregs. Overexpression of Mir-223-3p (148) and Protosappanin A intervention (149) can promote the phenotypic transformation from DCs to tDCs, induce Tregs generation, and inhibit cardiac inflammation and cardiac remodeling in EAM model mice. Of concern, EAM susceptibility differs between strains of mice. Compared to B10.S mice, A.SW mice have a lower ratio of Tregs *in vivo*, enhanced Th17 cell responses, greater sensitivity to autoimmunity, and more severe disease development in EAM (150).

### 5.5 Viral myocarditis

Intervention methods such as IL-37 (151) and Valproic acid (152) promote Th17/Treg immune balance and play an anti-inflammatory role, ameliorating CVB3-induced VMC. Cardiac Myosin peptide treatment and OX40 blockade (153), Fasudil (154), and nicotine (112) interventions improved cardiac inflammation and reduced mortality in CVB3-induced VMC mice by enhancing Tregs function. Adoptive transfer of Tregs can regulate TGF-β-Coxsackie-Adenovirus Receptor Pathway (155), promote monocyte differentiation into Ly6C^low^CCR2^low^CX3CR1^high^ subgroup with anti-inflammatory properties (156), enhance IL-10 secretion (157), and ameliorate cardiac function, inflammatory injury, and myocardial fibrosis in CVB3-induced VMC mice. B-cell deficiency can significantly reduce Tregs, damage Tregs’ immunosuppressive function, and damage myocardial Tregs homeostasis in CVB3-induced VMC mice, whereas adoptive transfer of B cells reverses this phenomenon (158). Latency associated peptide (LAP) is a membrane protein of Tregs. Compared with total Tregs, LAP^+^Tregs have greater immunomodulatory effects and may serve as a better VMC biomarker (159). In addition, *Astragalus Mongholicus (Fisch.) Bge* intervention improved cardiac function and peripheral Tregs immune imbalance in children with VMC by reducing miRNA-146b and miRNA-155 levels (160). The release of sex hormones and/or other mediators from the testis inhibits the population of anti-inflammatory cells in the heart, including Tregs, leading to more severe acute myocarditis with CVB3 infection in male mice (161). However, the adoptive transfer of M2 macrophages promoted peripheral Tregs differentiation and reduced cardiac inflammation in CVB3-induced VMC model male mice (162).

### 5.6 Chronic Chagas disease cardiomyopathy

Tregs are subsets of anti-inflammatory T cells with immunosuppressive functions that help limit tissue damage associated with an immune response triggered by the parasite (163). The mechanism by which immunotherapy with tDCs inhibits the progression of cardiac inflammation and myocardial fibrosis in a mouse model of CCC involves the secretion of IL-10 by tDCs to induce Tregs differentiation and enhance Tregs immunosuppressive function (164). IL-10 is a cytokine that can independently induce Foxp3 expression and Treg differentiation (165), and secretion of IL-10 by tDCS induces Foxp3^+^Tregs differentiation to regulate immunity (166, 167). Intervention with human recombinant granulocyte colony-stimulating factor (G-CSF) enhances cardiac Tregs recruitment and reduces cardiac inflammation, fibrosis, and parasite load in mice with CCC induced by chronic *T.cruzi* infection (168). Moreover, during the acute phase of *T.cruzi* infection, depleting Tregs exacerbated myocardial inflammation and tissue parasite levels, leading to increased mortality in experimental mice (169). In comparison, formyl peptide receptor 2 (FPR2) KO mice had increased Tregs during the acute *T.cruzi* infection phase, which controlled the protective effect of Th1 cells against *T.cruzi* infection. However, FPR2-KO mice have reduced Tregs and exacerbated cardiac inflammation and cardiac dysfunction during prolonged chronic *T.cruzi* infection (170).

### 5.7 COVID-19-associated myocarditis

A retrospective cohort study of 56963 hospitalized patients with COVID-19 showed that the incidence of acute myocarditis in hospitalized patients with COVID-19 ranged from 0.24 to 0.41%; Chest pain and dyspnea symptoms were the most frequent, accounting for 55.5% and 53.7%, respectively; 38.9% presented with fulminant manifestations; The combined incidence of in-hospital mortality or temporary mechanical circulatory support was 20.4%; At 120 days, the mortality rate was approximately 6.6% (171). Another retrospective cohort study involving 718365 COVID-19 patients showed that the incidence of COVID-19 with myocarditis and 6-month all-cause mortality were 5.0% and 3.9% respectively (172). Although COVID-19-associated myocarditis is very severe, the role of Tregs in it remain understudied.

## 6 Tregs regulate cardiac remodeling in CVDs

Cardiac remodeling is defined as changes in the size, shape, and function of the heart resulting from pathological conditions (173). Myocardial fibrosis is a qualitative and quantitative change in the myocardial interstitial collagen network characterized by excessive deposition of collagen and other extracellular matrix components. In ischemic heart disease, myocardial fibrosis exacerbates cardiac remodeling, promoting cardiac insufficiency, arrhythmias, and ultimately HF (174, 175). Targeted regulation of myocardial fibrosis and improvement of cardiac remodeling are effective therapeutic strategies for ischemic CVDs (176). T lymphocytes play an essential role in regulating extracellular matrix components and myocardial fibrosis by regulating the expression of myocardial collagen and matrix metalloproteinases, and the role of Tregs in myocardial fibrosis has also received attention (177, 178).

### 6.1 Myocardial infarction

Studies have shown that the adoptive transfer of Tregs inhibits myocardial fibrosis and cardiac remodeling in MI model animals (179, 180). Tregs can inhibit myocardial fibrosis and improve cardiac remodeling by regulating cardiac fibroblast phenotypes, reducing α-smooth muscle actin (α-SMA) expression, and extracellular matrix collagen deposition (181). Overexpression of Sparc enabled Treg to have a tissue repair phenotype, which helped to improve collagen content and maturity in scars after MI, prevent heart rupture, and improve MI survival rate (182). MI model mice CCR5^+^ monocytes promote the secretion of anti-inflammatory factor IL-10, mediate Tregs recruitment, inhibit inflammation, and inhibit myocardial fibrosis and cardiac remodeling. However, the expression of cardiac proinflammatory factors in CCR5^-/-^ MI model mice was significantly up-regulated, Tregs recruitment was impaired, and cardiac remodeling continued to worsen (183). IL-2/JES6-1 mAb (JES6-1) complex can improve cardiac function and remodeling by increasing the ratio of Tregs in MI model mouse heart infarct zone, inhibiting inflammation, inducing macrophages to transform from M1 to M2 type (184). Transferred myosin heavy chain α (MYHCA)_614–629_-specific CD4^+^T cells selectively accumulated in the myocardium and mediastinal lymph nodes of infarcted mice, acquired Tregs phenotype with a distinct pro-healing gene expression profile, and accelerated the regression of inflammation, promoted proper extracellular matrix deposition in the myocardial scar, and mediated cardioprotection (185).

### 6.2 Hypertension

Single-cell sequencing analysis of cardiac CD45^+^ immune cells in transverse aortic constriction-induced non-ischemic, pressure-overload HF model mice revealed that Tregs were activated, and the Tregs-specific molecule PD-1 was upregulated (186). Adoptive transfer of Tregs significantly ameliorated ventricular remodeling and myocardial fibrosis in rats with abdominal aortic constriction-induced HF by suppressing LOX expression *via* activation of the IL-10/Jak1/STAT3 signaling pathway (77). β-hydroxybutyrate can down-regulate NOX2/glycogen synthase kinase-3β (GSK3β) pathway, increase the number of cardiac Tregs, inhibit inflammation, and improve cardiac function, myocardial fibrosis, and cardiac remodeling in heart failure with preserved ejection fraction (HFpEF) mice (187).

Adoptive transfer of Tregs significantly reduced the infiltration of cardiac macrophages in Ang II-infused hypertension mice, improved cardiac inflammation, myocardial hypertrophy, and fibrosis, and inhibited electrical remodeling. The mechanism involved Tregs fixation of connexin 43 (CX43) gap junction protein in intercalated disk regions rather than lateral borders of cardiomyocytes, and reduced the risk of ventricular arrhythmias (188). Tregs with Nox2 deficiency by adoptive transfer significantly inhibited Ang II-induced hypertension and cardiac remodeling, and the effect was better than Tregs (60). In galectin-3 (Gal-3) KO hypertensive model mice, spleen Tregs significantly increased, and cardiac inflammation and myocardial fibrosis were improved (189). Overexpression of developmental endothelial locus-1 (DEL-1) in endothelial cells, combined with recombinant DEL-1 intervention, stabilized the number of α_v_β_3_ integrin-dependent Tregs and Il-10 levels, and improved cardiovascular remodeling and blood pressure levels in Ang II and DOCA-salt-induced hypertension mice (190). Tregs-derived IL-35 had a protective effect on right ventricular systolic pressure and right ventricular dilation in mice with pulmonary hypertension (191). IL-2/JES6-1 complex intervention effectively induced splenic Tregs amplification five times and inhibited Ang II mediated aortic collagen remodeling and atherosclerosis (192).

### 6.3 Atrial fibrillation

The abundance of *Bacteroides Fragilis* decreased in elderly patients with atrial fibrillation. *Bacteroides Fragilis* intervention can reduce the inflammatory response of aging rats by increasing the number of Tregs, inhibiting atrial remodeling, and preventing the occurrence of atrial fibrillation (193). Foxp3 is the direct target gene of miRNA-210. IL-6 promotes the expression of miRNA-210 by regulating HIF-1α, and inhibits Tregs function by targeting Foxp3, promoting myocardial fibrosis and exacerbating atrial fibrillation (194).

## 7 Tregs regulate plaque regression in atherosclerosis

Traditionally, atherosclerosis is considered to be a cholesterol storage disease caused by the retention of lipoproteins (including low-density lipoprotein, LDL) in the intimal space of arteries. The residual LDL is modified and absorbed by scavenger receptor-mediated phagocytosis, resulting in the continuous growth of fatty infiltration rich in inflammatory white blood cells and the formation of plaque. Plaque regression is an important clinical goal in the treatment of atherosclerosis. The increase of Tregs in plaque is one of the characteristics of plaque regression. The CD45^+^ cells isolated from aortic arch plaques of atherosclerotic mice were sequenced by single-cell RNA-sequencing, and the expression profiles of Tregs in progressing and regressing plaque were compared. The results showed that the Tregs in progressing plaques had high mRNA levels of thymus-derived or natural Tregs (nTregs) markers Nrp1 and nTregs-activated genes (Itgb1, CTLA4). In contrast, the level of Tregs Nrp1 mRNA in regressing plaque is lower, and the level of mRNA related to the differentiation or maintenance of Tregs is higher (Mif, lgals9, Ly6a), suggesting that Tregs in regressing plaque may come from the peripheral differentiation of naïve T cells (136). Under atherosclerotic pathological conditions, CX3CL1 was selectively recruited to the aortic wall, while CCL4, CXCL11 and CXCL9 mainly increased in lymph nodes. Although CX3CR1 was not significantly expressed in CD4^+^ T cells, overexpression of CX3CR1 in Tregs showed that the CX3CL1/CX3CR1 axis selectively chemotactic Tregs to the aortic plaque of atherosclerotic mice, reducing lipid deposition, increasing the content of collagen and smooth muscle cells to improve plaque stability, reducing the number of proinflammatory macrophages, and inhibiting the progression of atherosclerosis (195). Anti-CD3 antibody (CD3-Ab) significantly induced the rapid regression of plaque in the treatment of atherosclerosis. The mechanism is that CD3-Ab significantly reduced the infiltration of macrophages and CD4^+^ T cells in plaque and increased the proportion of Tregs in plaque. However, when the anti-CD25 antibody eliminates the function of Tregs, CD3-Ab cannot induce the regression of atherosclerotic plaque (196).

## 8 Tregs regulate immune tolerance

Heart transplantation is the only solution for end-stage HF, but it is limited by allogeneic heart rejection. One of the important pathophysiological processes of rejection after transplantation is inflammatory cell infiltration. Tregs mediate immune tolerance and regulate the immune microenvironment after heart transplantation.

Tregs-targeted Nox2 gene deletion (Nox2^fl/fl^Foxp3^Cre^) mice received allogeneic heart transplantation. Nox2-deficient Treg expressed higher levels of CCR4 and CCR8, driving Tregs to migrate to the transplanted heart and enhancing immunosuppressive function. Reduce the degree of cardiomyocyte necrosis and fibrosis in cardiac grafts (197). IL-34 is an inhibitory Tregs-specific cytokine as well as a tolerance cytokine, which can effectively inhibit allogenic reactive immune response and mediate transplant tolerance (198). The orthogonal IL-2/IL-2R system was used to target Tregs and selectively amplify Tregs to improve cardiac allotransplantation and enhance immune tolerance (199). Low-dose IL-2 can prolong the survival period of chronic cardiac allograft rejection model mice, increase the infiltration of CD4^+^CD25^+^Foxp3 Tregs in spleen and graft, increase the percentage of circulating FoxP3^+^PD-L1^+^exocrine and FoxP3^+^CD73^+^exocrine, and delay the rejection (200). Simvastatin combined with aspirin can activate Tregs to enhance immune tolerance, enhance the protective effect of vascular endothelial cells, and prolong the survival time of cardiac allograft (201). Sirtinol combined with FK506 has a synergistic effect on prolonging cardiac allograft survival, which regulates Th17/Treg balance by down-regulating IL-17A and up-regulating Foxp3 (10). In addition, a clinical study of 91 heart transplant patients showed that a low peripheral Treg/endothelial progenitor cell ratio after heart transplantation was an independent predictor of acute immune rejection (202).

Knockdown of circFSCN1 induced DC transformation into tDC phenotype, which contributed to Tregs amplification, prevented immune rejection of heart transplantation, prolonged allograft survival time, and reduced allograft fibrosis (203). Overexpression of growth differentiation factor 15 (GDF15) in DC enhances effector T cells depletion and promotes Tregs generation through the IDO signaling pathway, thus inhibiting immune rejection in cardiac allograft (204). The combination of marine and tacrolimus inhibited DC maturation through the reactive oxygen species (ROS)/ERK/NF-κB pathway, increased the rate of Tregs, reduced oxidative damage and apoptosis, and alleviated acute rejection of mouse heart allograft (205).

## 9 Targeted Tregs in the treatment of the neonatal cardiac injury

The neonatal mouse heart was injured from postnatal day (P) 0-7, and Tregs were recruited to directly promote myocardial cell proliferation and cardiac regeneration through paracrine CCL24, growth arrest specific 6 (GAS6), or amphiregulin (AREG). Depleted Tregs aggravate cardiac fibrosis, while adoptive transfer of Tregs reduces fibrosis and enhances the proliferation of injury cardiomyocytes. Single-cell sequencing analysis showed that there was no difference in Tregs transcriptomes whether neonatal hearts were regenerated or not, suggesting that adult Tregs had the same regenerative capacity as long as they were abundant (206). There were significantly more Tregs in the P8 hearts of newborn mice than in the first week after injury (207).

## 10 Discussion

**Figure 2:**
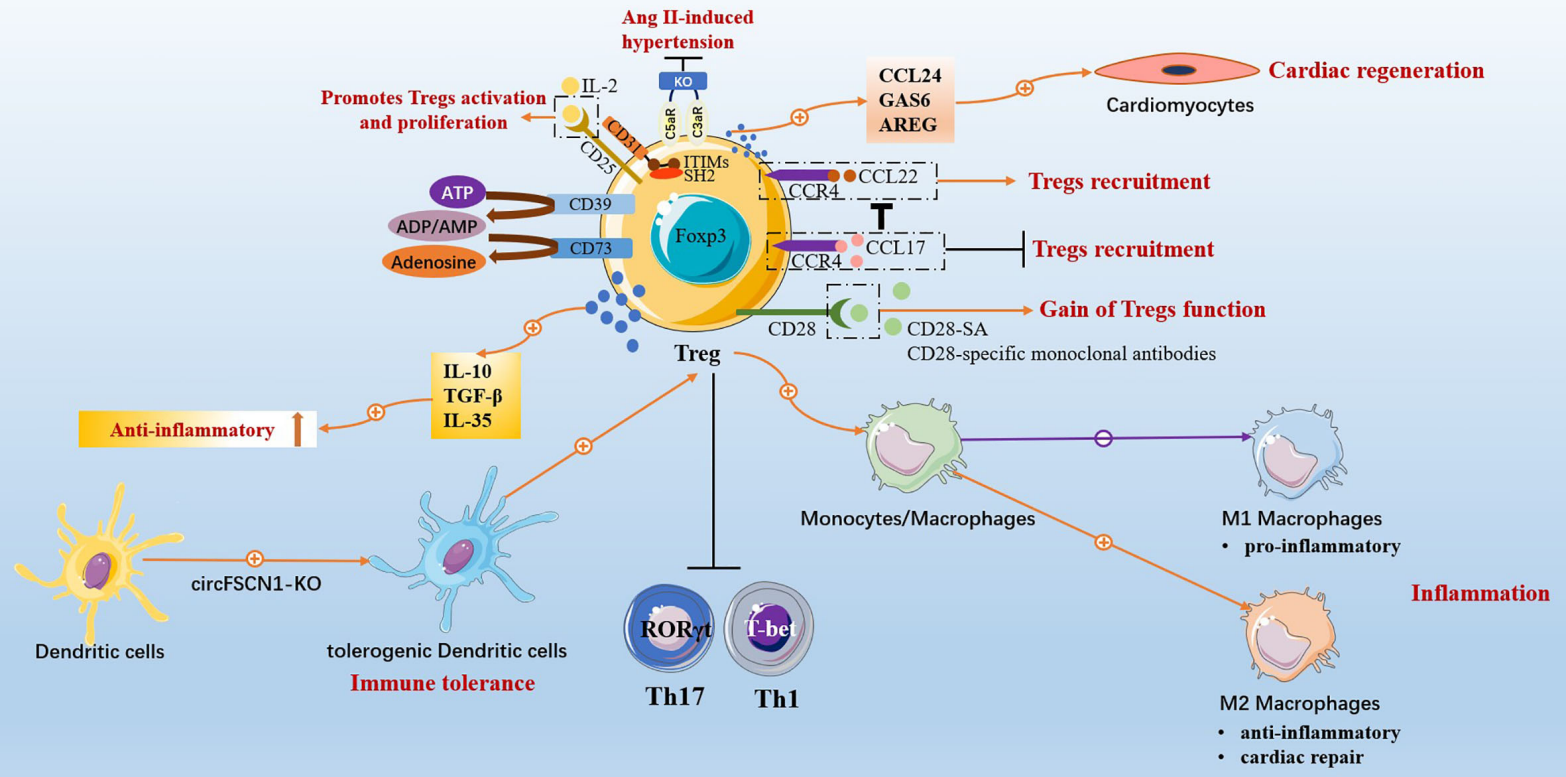
Protective effects of Conventional CD4^+^CD25^+^Foxp3^+^Tregs in cardiovascular diseases.

This review summarized that targeted Tregs effectively treat CVDs and have cardiac protective effects on MI, HF, myocarditis, hypertension, atherosclerosis, atrial fibrillation, heart transplantation, and neonatal heart injury. The specific mechanism involved Tregs regulating immune balance, anti-inflammatory, inhibiting cardiac remodeling and vascular remodeling, mediating immune tolerance, and promoting tissue regeneration and repair (**Figure 2**). Tregs inhibit the inflammatory response mediated by effector T cells, Th17 being the most significant, and regulate Th17/Treg to promote immune balance. Tregs regulate fibroblast phenotype and inhibit myocardial fibrosis and cardiac remodeling. Tregs promote the M2-type polarization of macrophages, which has anti-inflammatory and repair effects, and inhibit the M1-type polarization of macrophages, which has pro-inflammatory effects, thus enabling the recovery of damaged myocardium. Related intervention methods can promote Tregs amplification, enhance the immunosuppressive function of Tregs and further strengthen immune tolerance by regulating the transformation of DCs into tDCs phenotype. Tregs promote the regeneration of heart muscle cells and the repair of damaged hearts.

Conventional CD4^+^CD25^+^Foxp3^+^Tregs have a wide range of benefits in the treatment of CVDs. However, studies have shown the plasticity of Tregs (208), and this plasticity of Tregs has cardiotoxic effects on CVDs. Atherosclerosis can shift Tregs from a protective CXCR3^+^Treg response to dysfunctional interferon (IFN) γ^+^Th1/Treg response, driving inflammation and worsening disease progression (209). Tregs in the MI-post HF model mice showed pro-inflammatory Th1 cell characteristics, losing immunomodulatory function, enhancing anti-angiogenesis, and promoting fibrosis. Tregs reconstructed after selective dysfunctional proinflammatory Tregs ablation showed a recovery of immunosuppressive ability (210). The discovery of Tregs’ protean function and phenotypic plasticity in chronic ischemic HF has caused a considerable dispute in the cardiovascular field due to its novelty, challenging the conventional view of the phenotypic stability of Tregs after myocardial injury (211, 212), and generated extensive academic reports (213). HF disease can be divided into ischemic HF and non-ischemic HF. The cardioprotective effect of Tregs in non-ischemic HF and ischemic disease MI has been reviewed previously. While in MI-induced ischemic HF mice model experiments, a dysfunctional pro-inflammatory Tregs phenotype emerged. So, does it mean that Tregs play a typical functional role in a specific animal model? In addition, in the mice model of right lower extremity ischemia induced by right femoral artery ligation, although Tregs had immunosuppressive functions to suppress ongoing inflammation, Tregs had anti-neoangiogenic effects, resulting in foot inadequate perfusion and reduced capillary density (214). Human peripheral blood Tregs have IL-17^+^/Foxp3^+^Tregs phenotype and retain immunosuppressive function, while inhibition of Tregs Foxp3 expression *in vitro* and driven by inflammatory microenvironment show plasticity of IL-17 secretion (215). IL-17^+^/Foxp3^+^Tregs exist in the inflammatory intestinal mucosa of patients with Crohn’s disease and exhibit the phenotype of secreting IL-17 (216). The levels of the Th17 plasticity of Tregs are elevated in patients with rheumatoid arthritis (217). In autoimmune arthritis disease, the inflammatory microenvironment induces Foxp3 instability, leading to the trans-differentiation of Tregs into pro-inflammatory Th17 cells phenotypes, accelerating synovial membrane damage (218). So, do IL-17+/Foxp3+Tregs phenotype exist in heart tissue? Or does Foxp3 instability have a similar toxic effect on cardiovascular disease? It is worthy of further exploration. Moreover, Tregs are controversial in the context of myocardial fibrosis. Although much literature has reported that Tregs ameliorate cardiac fibrosis, TGF-β is secreted by Tregs, TGF-β/Smads are key pathways in the induction of fibrosis (219). Of course, the specific mechanism awaits further exploration.

The global burden of CVDs is still increasing. Although Tregs are a crucial target for the treatment of CVDs, there is still a lack of evidence from a large number of clinical randomized controlled trials. A few clinical trials in patients with CVDs have focused on measuring Tregs’ number, ratio, and function as a biomarker of disease severity. However, in terms of improving CVDs, whether endogenous Tregs are added, or exogenous Tregs are injected to enhance Tregs function, many basic experimental studies and rigorous efficacy and safety assessments are still needed before they can be used in clinical trials. This review summarized the clinical trials and basic experimental studies of targeted Tregs for CVDs, laying a foundation for further research on Tregs.

## Author contributions

XW: writing, editing and review of the manuscript. QL, PC, and TZ: revised the article. YZ, TY, and WS: Assisted with literature search. HZ, HQ, and YYZ: designed the conception and figures. All authors contributed to the article and approved the submitted version.
